# Microbial Dynamics between Yeasts and Acetic Acid Bacteria in Kombucha: Impacts on the Chemical Composition of the Beverage

**DOI:** 10.3390/foods9070963

**Published:** 2020-07-21

**Authors:** Thierry Tran, Cosette Grandvalet, François Verdier, Antoine Martin, Hervé Alexandre, Raphaëlle Tourdot-Maréchal

**Affiliations:** 1 UMR Procédés Alimentaires et Microbiologiques, Université de Bourgogne Franche-Comté/AgroSup Dijon, Equipe Vin Alimentation Micro-organismes Stress (VAlMiS) Institut Universitaire de la Vigne et du Vin Jules Guyot, 2 rue Claude Ladrey, BP 27877, 21000/26, bd Docteur Petitjean, BP 87999, 21079 Dijon, France; cosette.grandvalet@u-bourgogne.fr (C.G.); rvalex@u-bourgogne.fr (H.A.); tourdot@u-bourgogne.fr (R.T.-M.); 2 Biomère, 14 rue Audubon, 75120 Paris, France; fverdier@jubiles.bio (F.V.); amartin@jubiles.bio (A.M.)

**Keywords:** kombucha, yeasts, acetic acid bacteria, interactions, symbiosis, sucrose hydrolysis, pellicle

## Abstract

Kombucha is a traditional low-alcoholic beverage made from sugared tea and transformed by a complex microbial consortium including yeasts and acetic acid bacteria (AAB). To study the microbial interactions and their impact on the chemical composition of the beverage, an experimental design with nine couples associating one yeast strain and one AAB strain isolated from original black tea kombucha was set up. Three yeast strains belonging to the genera *Brettanomyces*, *Hanseniaspora*, and *Saccharomyces* and three strains of *Acetobacter* and *Komagataeibacter* species were chosen. Monocultures in sugared tea were analyzed to determine their individual microbial behaviors. Then, cultivation of the original kombucha consortium and cocultures in sugared tea were compared to determine the interactive microbial effects during successive phases in open and closed incubation conditions. The results highlight the main impact of yeast metabolism on the product’s chemical composition and the secondary impact of bacterial species on the composition in organic acids. The uncovered microbial interactions can be explained by different strategies for the utilization of sucrose. Yeasts and AAB unable to perform efficient sucrose hydrolysis rely on yeasts with high invertase activity to access released monosaccharides. Moreover, the presence of AAB rerouted the metabolism of *Saccharomyces* *cerevisiae* towards higher invertase and fermentative activities.

## 1. Introduction

Fermented food is often designated as a convenient model for the study of microbial communities and interactions [1,2,3]. Kombucha is an increasingly exploited example of a fermented beverage obtained by microbial communities. Kombucha, also named “kombucha tea”, results from the metabolic interplay of a microbial consortium including acetic acid bacteria (AAB), yeasts, and often (but not always) lactic acid bacteria in sugared tea liquor [4,5,6]. Infusion provides nitrogenous substances extracted from tea necessary for the growth of microorganisms. Sucrose is converted into glucose and fructose by periplasmic yeast invertase and ethanol is produced as a result of alcoholic fermentation. AAB oxidize glucose into gluconic acid and ethanol into acetic acid through oxidative metabolism. This metabolic scheme raises the question of a possible trophic dependency of AAB towards yeasts [7]. Glucose and fructose are also used for bacterial cellulose production, leading to the formation of the pellicle, also known as “mother”, “tea fungus”, or Symbiotic Culture of Yeast and Bacteria (“SCOBY”), since it can be used as inoculum with or instead of liquid culture [4,5,8]. As a result, the beverage has the profile of a soda with a sweet/sour balance and can also be carbonated naturally if the vessel is left closed after a first phase of biological acidification at ambient temperature [9]. There is no single “culture” or microbial consortium for the production of kombucha, but a multitude of matrix-adapted consortia whose origins are unknown. This matrix also offers two distinct environments: A liquid phase, where microorganisms are in a planktonic state, and a pellicle, where they are entrapped. Biofilms are known to host numerous interaction mechanisms among microbial communities and the cellulosic pellicle produced by the AAB of kombucha is no exception [10,11,12]. Beyond this trophic interaction, little is known about other types of interactions that might occur during kombucha fermentation at genus or species levels. The formation of the pellicle, as well as the drop of pH, can work as protection against contamination by exogenous spoilage agents such as molds [13]. Inside the consortium, inter-kingdom and intra-kingdom interaction mechanisms remain poorly documented.

Some studies have provided clues on the existence of interactions through the lens of microbial dynamics. The results have shown that some bacterial species are dominant, but the dynamics are dependent on the type of tea used (black or green) [14]. It has also been shown that the population of some yeast species decreases during kombucha fermentation, while others remain stable, as was the case for *Torulaspora delbrueckii* or *Schizosaccharomyces pombe* in the presence of *Zygosaccharomyces bailii*. In this case, the impacted yeast species were dependent on the consortium and this phenomenon did not occur in pellicles, with all populations being maintained [15]. Microbial interaction data based on indigenous complex consortia are difficult to summarize because of the complexity of the original system. Focus has been placed on microbial dynamics in terms of populations, without investigating the impact on the chemical composition of the beverage. To the best of our knowledge, only one study has investigated the interactions between individual isolates from kombucha by cultivating AAB (*Acetobacter* sp.) in sugared black tea with the addition of autoclaved sugared black tea fermented by a single strain of yeast (*Saccharomyces cerevisiae*, *Brettanomyces bruxellensis*, and *Zygosaccharomyces bailii*) [16]. This study concluded on the positive impact of the addition of fermented medium on AAB growth and also suggested that acetic acid produced by AAB could stimulate the production of ethanol by yeast. A recent study compared an original kombucha consortium with an unknown microbial composition, and a synthetic consortium including purchased isolates of species commonly found in kombucha [17]. Two AAB species, consisting of *Acetobacter pasteurianus* and *Komagataeibacter xylinus*, were used with the yeast species *Z. bailii*. The study confirmed the technical suitability of using a synthetic consortium of yeasts and AAB for the fermentation of kombucha beverages and highlighted the metabolic interplay between yeasts and AAB.

The present study goes further in the study of the yeast–bacterium interaction that occurs during kombucha fermentation in terms of both microbiological levels and the composition of the main metabolites produced. Yeasts and bacterial strains were isolated from an original black tea kombucha culture, identified, and selected to develop an experimental design with three yeast strains and three AAB strains. Each yeast and AAB was characterized individually, co-inoculated (one yeast strain x one AAB strain, with “x” indicating a coculture) in sugared black tea, and compared to the original consortium. This methodology was inspired by the integrated design for the study of microbial communities described by Lawson et al. (2020), consisting of joint studies of the original consortium, with simplified and deconstructed consortia reassembled using isolates [18]. Moreover, on top of the aerobic acidification phase, the anaerobic carbonation phase in a closed bottle was investigated for the first time.

## 2. Materials and Methods

A diagram summing up the experiment is available as Supplementary Data (Figure S1).

### 2.1. Isolation and Identification of Microbial Species

Yeast and bacterial strains were isolated from liquid and pellicle samples of black tea kombucha from the company Biomère (Paris, France). WallersteinLab (WL) agar medium from Thermo Fisher Scientific (Waltham, MA, USA) was used to isolate yeasts [19]. Bromocresol Green allowed a macroscopic discrimination of colonies based on their aspect and color for the set of yeasts isolated in the present study [20,21]. De Man Rogosa and Sharpe (MRS) (pH 6.2) from Condalab (Madrid, Spain), LAC (pH 5.1), and Mannitol agar media [22] were used for the isolation of bacteria that may have different nutritional requirements in both aerobic and anaerobic conditions of incubation [23]. All reagents used in agar media were purchased from Merck (Darmstadt, Germany), if not otherwise specified.

Five yeast colonies per colony morphotype were separately picked-up for inoculation in Yeast Peptone Dextrose (YPD) liquid medium (48 h at 28 °C). Twenty-five bacterial colonies per agar medium were separately picked-up for inoculation in LAC medium (48 h at 28 °C in the aerobic condition of incubation), since all species identified on Mannitol agar medium were also identified on LAC agar medium. After incubation, culture media were eliminated by centrifugation (14.500× *g*, 3 min at 15 °C). A half volume of appropriate liquid medium with a half volume of 40% (v/v) glycerol was then added to the samples to make a stock kept at −20 °C.

DNA extraction from each isolated strain was performed using an Instagen Matrix kit (Bio-Rad, Hercules, CA USA). For yeasts, the 26S rDNA region ribosomal non transcribed spacer 2 (NTS 2) was amplified using the following primers: NL1 (5′-GCATATCAATAAGCGGAGGAAAAG-3′) and NL4 (5′-GTCCGTGTTTCAAGACGG-3′) [24]. Two microliters of extracted DNA were added to 25 µL PCR mix (1.8 mM MgCl_2_, 0.25 mM dNTPs, 1.25 µM of each primer, and 0.025 U Taq polymerase) (Promega Corp., Madison, WI, USA). A Biorad (Hercules, CA, USA) thermocycler was used as described previously [25].

For bacteria, 16S ribosomal DNA was amplified using the following primers: E517F (5′-GCCAGCAGCCGCGGTAA-3′) and E106R (5′-CTCACGRCACGAGCTGACG-3′). Amplification was performed in the same conditions as for the yeasts, except for the annealing temperature, which was changed to 58 °C [26]. Both strands Sanger sequencing was performed on amplified DNA by Genewiz^®^ (Leipzig, Allemagne) using NL1 and NL4 primers for yeasts and E515F and E106R primers for bacteria. The sequences obtained were then analyzed using the software Geneious R7 (version 7.1.5) and the Basic Local Alignment Search Tool (BLAST) available on NCBI’s website (https://blast.ncbi.nlm.nih.gov/Blast.cgi), thus returning genus and species names.

### 2.2. Growth Conditions

In order to generate pure precultures, yeasts were restreaked on WL agar and bacteria were restreaked on MRS agar from stocks kept at −20 °C and incubated at 28 °C. MRS agar was chosen because it is a commonly used growth medium for exigent bacteria, and the growth of each isolated AAB species on this medium was tested prior to the experiment.

Cultivation of the original kombucha consortium and cocultures was initiated in sugared tea. To produce 1 L of sugared black tea, tap water was filtered through a Brita (Taunusstein, Allemagne) charcoal cartridge and sterile filtered by a Steritop^®^ cartridge by Merck (Darmstadt, Allemagne). Then, 200 mL of sterile tap water was boiled and directly taken off the heating source. One gram of black tea (Pu’er Grade 1 TN4107 from “Les jardins de Gaïa”) (Wittisheim, France) was immediately added and left for infusion for one hour at room temperature. Tea was then removed using a sieve and transferred to a sterile vessel. A total of 800 mL of sterile tap water at 26 °C was added. Then, 60 g sucrose was added and dissolved completely. The mix was kept at 26 °C in a closed Schott^®^ flask for one hour, prior to inoculation. Yeast and AAB strains were cultivated in YPD and MRS liquid medium, respectively. The tubes were not fully closed, in order to allow gaseous exchange during the incubation at 28 °C in static conditions for 3 days. Before inoculation in sugared tea, cells from pure cultures were washed with sugared black tea and then centrifugated (3.000× *g*; 10 min at 4 °C). The populations of pure cultures were determined using a BD Accuri C6 (Franklin Lakes, NJ, USA) flow cytometer coupled with 0.1 µg mL^−1^ propidium iodide marking for cell mortality [27].

The choice of the target inoculation rate for individual species was based on the population levels determined for original kombucha at the inoculation time. Consequently, sugared black tea was then inoculated using precultures with an initial population of yeast or AAB of 1.10^5^ cells mL^−^^1^. For cocultures, the same process as that employed for pure cultures was used, with initial populations of 1.10^5^ cells mL^−1^ for both yeast and bacterium strains.

The production of kombucha was carried out using a kombucha mother culture maintained in the lab from the original black tea kombucha sample from Biomère (Paris, France) by adding a half volume of sugared black tea each month. Kombucha mother culture was added to sugared black tea at the rate of 12% (v/v) and kept at 26 °C for 14 days in a 500 mL Schott^®^ bottle, in order to produce a fresh kombucha inoculum. 12% of this inoculum was added to fresh sugared black tea to produce the traditional kombucha used in this study.

Monocultures, cocultures, and original kombucha incubations were performed in 125 mL Boston flasks with a Specific Interfacial Surface (SIS) of 0.01 cm^−1^ in a final volume of 123 mL [28]. For monocultures in an open incubation condition (IC), bottlenecks were loosely covered with tin foil to allow gas exchange, whereas for closed IC, flasks were closed using a cap to reflect bottling. Monocultures were characterized in both open and closed IC at 26 °C after 7 and 14 days for microbiological analysis and after 14 days for chemical analyses. Cocultures and kombucha incubations were carried out in two phases. The first phase (P1) of fermentation was performed for 14 days at 26 °C in the same open IC as monocultures. Then, half of the volume was withdrawn for analysis (“P1” samples). The bottles were then sealed, in order to trigger natural carbonation. This phase (P2) was performed at 26 °C for 10 days. At the end, the samples were analyzed (“P2” samples). Open and closed IC do not strictly reflect aerobic or anaerobic systems, but rather the production process of kombucha making in industrial conditions. Dissolved oxygen concentrations were not measured in samples. A description of the research samples and abbreviations is detailed as Supplementary Data (Table S2).

The populations of yeasts and/or bacteria during fermentation were determined by plating successive decimal dilutions of samples on WL agar for yeasts and MRS agar for bacteria, with technical triplicates for each biological triplicate.

### 2.3. Chemical Analyses

For chemical analyses, samples were kept frozen at −20 °C and centrifuged prior to chemical analyses (3.000× *g*; 15 min, 10 °C). pH values were measured with a Mettler Toledo Five Easy pH meter coupled with an LE498 probe. The total acidity was determined by titration with 0.1 N NaOH and 0.2% phenolphthalein as a color indicator [29].

Acetic, lactic, malic, and succinic acid concentrations were determined by HPLC with a VWR Hitachi (Tokyo, Japan) control unit, L-2350 oven, L-2200 autosampler injection device, and L-2130 pump. The column used was a Raptor ARC-18 2.7 µm 150 × 2.1 mm from RESTEK (Lisses, France) and the detector was a UV Diode Array Detector VWR Hitachi L-2455 (Tokyo, Japan) at 210 nm. The mobile phase was 97% 20 mM KH_2_PO_4_ (pH 2.4) and 3% methanol. A flow gradient was applied at 35 °C, with the following sequence for a total duration time of 10 min: From 0.1 mL min^−1^ to 1.0 mL min^−1^ for 5 min, 1.0 mL min^−1^ for 3 min, and 0.1 mL min^−1^ for 2 min.

Sucrose, glucose, and fructose concentration determinations by HPLC involved the same equipment, except for the column. A HyperREZ XP Carbohydrate Ca++ 8% column from Thermofisher (Waltham, Etats-Unis) was used with a Spectrasystem RI-150 refractometer from de JMBS (Mandelieu-Napoule, France). The flow rate was 0.6 mL min^−1^ with ultrapure water at 80 °C.

Gluconic acid and ethanol concentrations were determined using enzymatic kits from Biosentec (Auzeville-Tolosane, France).

The free amino nitrogen (FAN) concentration was determined according to the protocol from MEBAK^®^ (2013) [30], with the following adaptation. In total, 0.4 mL 20-fold diluted sample was added to 0.2 mL reactive mix (0.71 M Na_2_HPO_4_, 0.44 M KH_2_PO_4_, 28 mM ninhydrin, and 17 mM fructose). The mix was heated for 16 min at 100 °C and left to cool down for 20 min at room temperature. Then, 0.8 mL dilution solution (40% (v/v) ethanol, 12 mM potassium iodide) was added before the absorbance reading at 570 nm using a UV-1800 spectrophotometer from Shimadzu (Kyoto, Japan). Distilled water was used as the blank. A calibration curve was made using a glycine solution.

Analytical chemistry results were expressed as the balance, according to the following formula (1):

∆Concentration = ∆Endpoint concentration − ∆Initial concentration.(1)

Initial corresponds to day 0 and the “endpoint” corresponds to day 14 for monocultures and “P1” or “P2” for cocultures.

### 2.4. Statistical Analyses

All samples were made in triplicate. Values were treated with ANOVA and in the case of significant differences (*p* < 0.05), the Newman–Keuls pair test was applied. Principal Component Analysis (PCA) coupled with Hierarchic Ascending Classification (HCA) was also performed for P1 and P2 values separately. All statistical analyses were performed with R software (version 3.5.2.).

## 3. Results and Discussion

### 3.1. Isolation and Identification of Yeast and Bacterial Strains

Based on the colony morphology on WL agar, yeast species in the original black tea kombucha sample were isolated and then identified by DNA sequencing of the 26S ribosomal region [24]. In the liquid phase, *Brettanomyces* (*Dekkera*) *bruxellensis* (small white colonies), *Hanseniaspora valbyensis* (dark green colonies), and *Saccharomyces cerevisiae* (large white colonies) were identified. In the biofilm, the same species were isolated, in addition to *Hanseniaspora opuntiae* (bright green colonies), *Pichia aff. fermentans* (white star-shaped colonies), and *Galactomyces geotrichum* (white filaments). Yeast colony morphologies were all distinct for each species. On agar plates, all AAB colonies had a sticky and translucid aspect. DNA sequencing of the 16S ribosomal region [26] allowed the identification of the species *Acetobacter indonesiensis*, *Acetobacter papayae*, and *Komagataeibacter saccharivorans*, which were all isolated in both liquid and biofilm phases. All species names were associated with E-values equal to 0. No lactic acid bacteria were identified, despite the use of LAC medium in both aerobic and anaerobic conditions of incubation. Except for *Galactomyces geotrichum*, the genera of isolated yeasts and bacteria are commonly found in kombucha [4]. For this study, species found in the liquid phase were chosen because they were present in both liquid and biofilm. Each of the selected strains was characterized in sugared black tea.

### 3.2. Characterization of Pure Cultures in Sugared Black Tea

**Figure 1:**
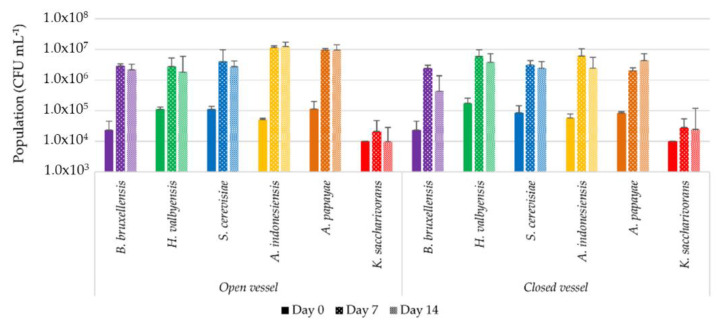
Microbial populations during cultivation in sugared black tea of a monoculture of yeast and bacterial strains isolated from black tea kombucha determined by plate counting (CFU mL^−1^). Error bars correspond to the confidence interval with α = 0.05 (*n* = 3). Cultures were conducted in open vessel (left) or closed (right) conditions of incubation for 14 days.

Figure 1 presents the population levels of each selected strain inoculated in sugared black tea after inoculation on day 0 and after incubation for 7 days and 14 days at 26 °C in open and closed incubation conditions (IC).

Yeast population levels increased from 1.0.10^5^ ± 5.10^4^ CFU mL^−^^1^ to values between 1.0.10^6^ CFU mL^−1^ and 6.0.10^6^ CFU mL^−^^1^, regardless of the open or closed IC after 7 days. These levels of population were maintained 7 days later.

Except for *K. saccharivorans*, AAB’s population levels increased from 1.0.10^5^ ± 5.10^4^ CFU mL^−^^1^ to values between 1.0.10^6^ CFU mL^−1^ and 1.0.10^7^ CFU mL^−^^1^, regardless of the open or closed IC after 7 days. It can be supposed that, on top of assimilable nutrients, the medium initially possessed a sufficient dissolved oxygen level to allow the growth of AAB in closed IC. These levels of population were maintained 7 days later. *K. saccharivorans* possessed a lower population at day 0 and the population only increased by less than 1 log. Discrepancies between the target inoculation rate and plate counting results at day 0 could be due to the viable but not culturable (VBNC) state, as reported for *Komagataeibacter xylinus* [31,32].

The average initial composition of sugared black tea was 66.6 ± 3.3 g L^−^^1^ total sugars (66.4 g L^−^^1^ sucrose, 0.2 g L^−^^1^ fructose, and no glucose detected). The FAN concentration was 63 ± 4 µg L^−^^1^, which is very low compared to grape must, for example (between 50 and 150 mg L^−^^1^) [33]. The initial average pH value was 6.64 ± 0.47 units and average total acidity was lower than 1 meq L^−1^. No organic acids nor ethanol were detected. The chemical composition variation of sugared black tea after the incubation of each culture (day 14) is detailed in Table 1.

For yeasts, the decrease of total sugars indicated a maximal sugar variation of −10.7 g L^−^^1^. No groups could be formed among the different values, despite significant differences (*p* < 0.05). Sucrose underwent variations from null to total disappearance (−68.3 g L^−^^1^), with significant differences between the values (*p* < 0.05). The monosaccharide content varied significantly from 0.0 to 28.5 g L^−^^1^ for glucose (*p* < 0.05) and from 0.0 to 31.8 g L^−^^1^ for fructose (*p* < 0.05). Their production succeeded sucrose hydrolysis. A significant production of ethanol was also observed, with variations between 0.1 and 3.2 from 0.0 to 28.5 g L^−^^1^ (*p* < 0.05), which is representative of the fermentation activity. The results show very different sugar consumption behaviors across the three yeasts species and highlight the link between invertase activity, respiration, and fermentation. *B. bruxellensis* is characterized by high invertase activity and fermentative metabolism in both open and closed IC. For this strain, ethanol production was significantly higher in open IC than closed IC (*p* < 0.05). The inhibition of alcoholic fermentation under anaerobic conditions is called the Custer effect and was reported for *B. bruxellensis* [34,35]. *H. valbyensis* is characterized by poor sucrose hydrolysis and fermentative capacities, particularly in open IC. The very low consumption of sucrose was reported in the context of cider production [36]. The same applied to *S. cerevisiae* in open IC, but the presence of invertase activity is more evident and points to the induction of *SUC* genes in the presence of a low glucose concentration [37,38]. This mechanism allows *S. cerevisiae* to progressively hydrolyze sucrose and consume monosaccharides, without inducing catabolic repression by glucose. However, oxygen limitation in closed IC induced fermentation and increased sugar consumption (Pasteur effect), as reported by the data summarized in Marques et al. (2016) [39]. According to this review, the increase in sugar consumption may be a consequence of the lower energetic yield of fermentative metabolism compared to respiration. Then, it can be supposed that the low sucrose consumption observed in *S. cerevisiae* monoculture in open IC is associated with respiratory metabolism, which could also be the case for *H. valbyensis*. In addition, slightly lower glucose concentrations could be observed for modalities associated with fermentative metabolism. Studies have reported preferential glucose consumption during the alcoholic fermentation of grape must [40,41]. The free amino nitrogen (FAN) concentration increased in all modalities between 3 and 54 µg L^−1^ and it also seems to be enhanced by fermentative metabolism induced by oxygen limitations, although a significant increase between open and closed IC was only observed for *S. cerevisiae* (*p* < 0.05).

The increase in total acidity (between +1 and +8 meq L^−^^1^) led to a decrease of the pH value, ranging from −1.7 to −2.7 units. This increase in total acidity was significantly more intense for *B. bruxellensis* compared to *S. cerevisiae* and the increase for *S. cerevisiae* was also more intense than for *H. valbyensis* in open IC (*p* < 0.05). *B. bruxellensis* is characterized by the production of acetic acid. Indeed, this species is strongly associated with the production of this organic acid [34]. For the whole set of yeasts, the accumulation of succinic and malic acid and the absence of lactic acid production seem to be linked to fermentative metabolism in these conditions.

Despite the initial absence of monosaccharides added, AAB were able to consume between 0.7 and 10 g L^−^^1^ total sugars. The presence of monosaccharides after 14 days points to the hydrolysis of sucrose in the external medium. This phenomenon has been reported in several studies [17,42,43,44]. The total acidity increased between +5.7 and +19.3 meq L^−1^ and induced a drop of pH between −2.05 and −2.64 units, with significant differences between the values (*p* < 0.05). The production of gluconic acid from the oxidation of released glucose between +0.11 and +1.94 g L^−^^1^ was significantly lower with closed IC for all AAB species except *A. papayae* (*p* < 0.05) and could be explained by the limitation of oxygen [7]. The production of acetic acid between +0.25 and +0.56 g L^−^^1^ without the initial presence of ethanol can be explained through the glycolysis and pyruvate metabolism of AAB [44,45,46] and has been reported in the study of Wang et al. (2020) [17]. Succinic acid production occurred between +0.14 and +0.16 g L^−^^1^ for *K. saccharivorans* monocultures and malic acid production only occurred for *Acetobacter* sp. cultures in closed IC. It is worth noting that no consistent biofilm was produced after 14 days in all conditions; only floating cellulose fragments were visible. Overall, AAB could hydrolyze sucrose and consume monosaccharides. Oxygen limitations in closed IC impacted oxidative metabolism converting glucose into gluconic acid, and thus a limited acidification of the medium.

The interpretation of coculture results in comparison with original kombucha fermentation on the basis of monoculture characterization will help clarify the impact of each metabolic profile on the yeast–AAB interactions.

### 3.3. Comparison of Yeast-Acetic Acid Bacteria Cocultures with Original Kombucha Fermentation

#### 3.3.1. Microbial Dynamics

The experimental plan for yeast-AAB cocultures and their abbreviations are presented in Table 2. These pairings can be seen as minimal kombucha consortia that allow the metabolic interplay necessary for the fermentation of kombucha. This process occurred at 26 °C in two phases: An initial 14-day phase in open IC and a second phase of 10 days in closed IC.

**Figure 2:**
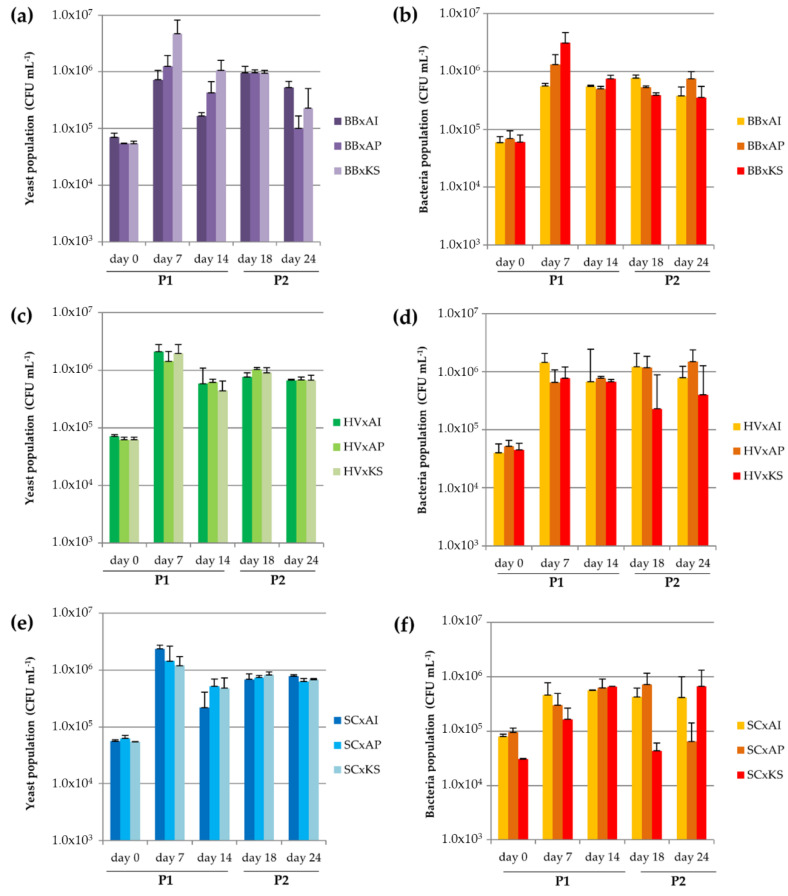
Microbial populations of yeast and bacterial cocultures in sugared black tea determined by plate counting (CFU mL^−^^1^). Error bars correspond to the confidence interval with α = 0.05 (*n* = 3). (**a**) and (**b**) Yeast and bacterial populations, respectively, in cocultures involving *Brettanomyces bruxellensis* (BB). (**c**) and (**d**) Yeast and bacterial populations, respectively, in cocultures involving *Hanseniaspora valbyensis* (HV). (**e**) and (**f**) Yeast and bacterial populations, respectively, in cocultures involving *Saccharomyces cerevisiae* (SC). AI = *Acetobacter indonesiensis*, AP = *Acetobacter papayae*, and KS = *Komagataeibacter saccharivorans*.

All yeast populations in coculture (Figure 2a,c,e) at day 14 remained significantly lower (1 to 0.5 log) than those in yeast monocultures at day 14 in open IC (Figure 1). This could mean that the presence of AAB lowered the population of yeasts, regardless of the species. Possible explanations could be nutritional competition [1] or inhibition through the production of acetic acid (see Table 3) [47,48]. AAB populations increased significantly above 1.10^5^ CFU mL^−^^1^ at day 7. Only cocultures BB x AP, BB x KS, and HV x AI (Figure 2b,d,f) reached bacterial populations beyond 1.10^6^ CFU mL^−^^1^ on day 7. Between day 14 and day 24, populations remained stable in the range between 1.10^5^ and 1.10^6^ CFU mL^−^^1^. The strongest variations could be seen with the modalities SC x AP and SC x KS, with populations below 1.10^5^ CFU mL^−1^. Populations of *Acetobacter* sp. in coculture at day 14 were lower than those in monocultures in open IC at day 14 by around 1 log. On the contrary, populations of *K. saccharivorans* were higher in cocultures by around 1.5 log.

**Figure 3:**
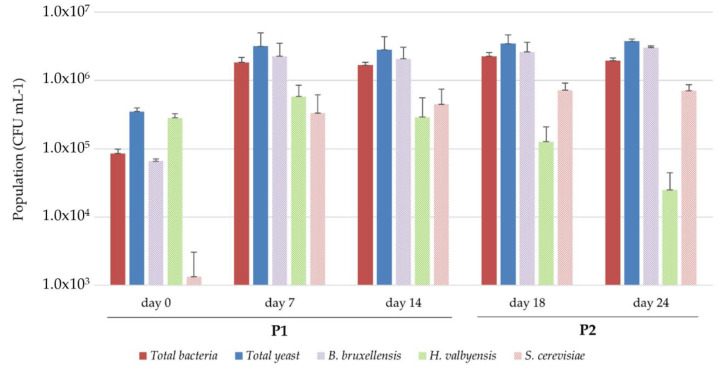
Microbial populations during cultivation in sugared black tea of the black tea kombucha consortium determined by plate counting (CFU mL^−^^1^). Error bars correspond to the confidence interval with α = 0.05 (*n* = 3).

During original kombucha fermentation, total yeasts and total bacterial populations at inoculation were 3.5.10^5^ ± 4.6.10^4^ CFU mL^−^^1^ and 8.5.10^4^ ± 1.3.10^4^ CFU mL^−^^1^, respectively (Figure 3). Yeast and bacterial populations increased to 3.2.10^6^ ± 1.8.10^6^ CFU mL^−^^1^ and 1.8.10^6^ ± 3.2.10^5^ CFU mL^−^^1^, respectively, and stayed stable until day 24. The total yeasts population thus remained slightly higher than the total bacteria population throughout the fermentation. This was not observed for cocultures. Both yeast and AAB populations were lower in cocultures than in original kombucha fermentation (Figure 2). The general higher population in original kombucha could indicate different microbial dynamics of the consortium compared to cocultures. The differences in the yeast/AAB balance could be explained by the absence of consistent pellicle formation in cocultures, with a fragile veil appearing instead. The AAB population present in the biofilm in original kombucha could then be found in the liquid phase of cocultures instead [49,50]. The different yeast colony morphotypes allowed a discrimination of populations by species. At the inoculation time, *H. valbyensis* was the major yeast species (6.6.10^4^ ± 5.1.10^3^ CFU mL^−^^1^), followed by *B. bruxellensis*. (2.8.10^5^ ± 4.3.10^4^ CFU mL^−^^1^). The *S. cerevisiae* population was clearly lower, with 1.3.10^3^ ±1.7.10^3^ CFU mL^−^^1^ (Figure 3). After 7 days, all populations increased beyond 5 log and *B. bruxellensis* became the major species, followed by *H. valbyensis* and then *S. cerevisiae*. The same levels of population were found at day 14. It is worth noting that these proportions differ in comparison to those of the inoculum (fermentation of 14 days) and consequently the sample at day 0 (inoculation). This could be explained by differences in oxygen access due to the greater SIS of the preculture compared to the SIS used during the experiment. Change occurred at day 18 and day 24 after closing the bottle, with a parallel decrease of the *H. valbyensis* population (−2.7.10^5^ CFU mL^−^^1^) at day 24 and an increase of *S. cerevisiae* (+1.8.10^5^ CFU mL^−^^1^). The *B. bruxellensis* population remained stable between day 7 and day 24, which highlights a predominant role during the whole process. The population dynamics induced by vessel closing were not observable in cocultures (Figure 2).

#### 3.3.2. Utilization of Carbohydrates

**Figure 4:**
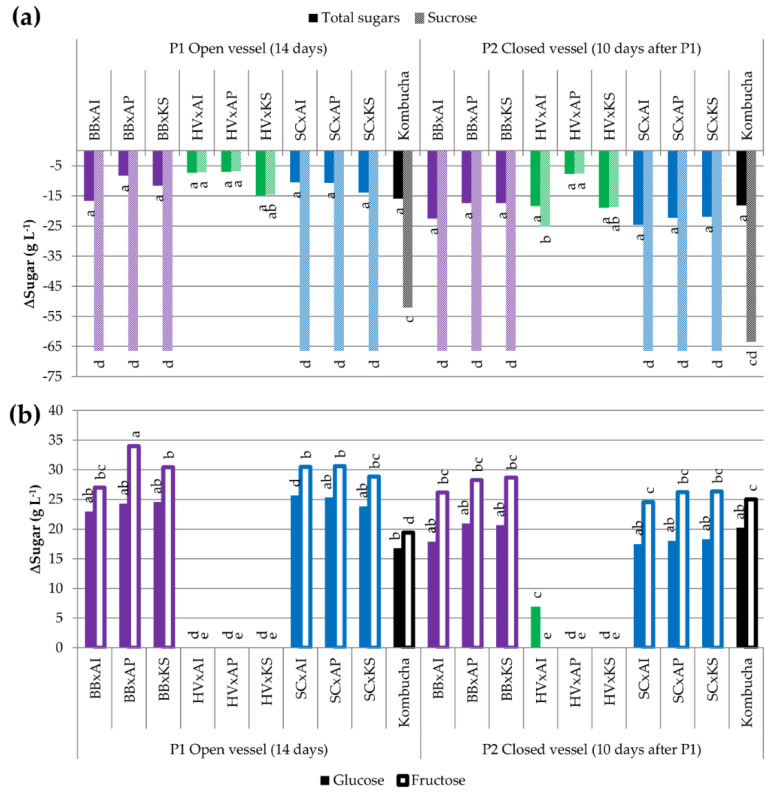
Difference in total sugars and sucrose (**a**) and in glucose and fructose (**b**) (g L^−^^1^) of samples between day 0 (after inoculation) and the end point (14 days for pure cultures, 14 days in an open vessel for P1, and 10 days in a closed vessel following P1 for P2). ANOVA was performed with α = 0.05 and *n* = 3. Common letters imply non-significant differences between means. Colors reflect the yeast species in the coculture. BB = *Brettanomyces bruxellensis* (purple), HV = *Hanseniaspora valbyensis* (green), SC = *Saccharomyces cerevisiae* (blue), AI = *Acetobacter indonesiensis*, AP = *Acetobacter papayae*, KS = *Komagataeibacter saccharivorans*, and “x” = a coculture.

No groups could be determined among the total sugar variation values, ranging between −5 and −27 g L^−^^1^, despite the presence of significant differences (*p* = 0.008) (Figure 4a). However, the average values of total sugar variations are all lower between the aerobic acidification phase (14 days after inoculation, P1) and anaerobic acidification phase (10 days in closed IC, P2). This means that most of the sugars were consumed during P1. The sucrose hydrolysis of cocultures, reflected by the disappearance of sucrose, was similar to the capacities observed by the corresponding yeast monoculture, except for *S. cerevisiae.* This species behaved closer to monocultures in the closed IC, with the complete degradation of sucrose during P1 (Figure 4a and Table 1). All cocultures except those including *H. valbyensis* achieved significantly stronger sucrose hydrolysis at P1 compared to kombucha (*p* < 0.05), but this gap was filled at P2 (Figure 3a). This highlights a strong impact of yeasts on the release of monosaccharides that are used by AAB. The results displayed in Figure 4-b show a significantly greater release of monosaccharides of *B. bruxellensis* and *S. cerevisiae* compared to *H. valbyensis*, thus confirming the release of glucose and fructose for cocultures including yeasts with strong known invertase activity (*p* < 0.05 for glucose and *p* < 0.05 for fructose). It is noteworthy that in all cases at P1, the fructose content was higher than glucose at P1 and P2, which was characteristic of yeast metabolism in monocultures (Table 1), but preference for glucose could also occur for AAB.

Original kombucha underwent an incomplete hydrolysis of sucrose at P1 (−52.0 g L^−^^1^ sucrose), but the process was completed at P2 (−63.4 g L^−^^1^ sucrose), although the total sugar consumption did not evolve significantly (−15.9 and −18.2 g L^−^^1^ total sugars at P1 and P2, respectively; the initial amount of total sugar was 72.5 g L^−^^1^ with 71 g L^−^^1^ sucrose and 1.5 g L^−^^1^ fructose, and glucose was not detected). It can be supposed that the conditions in original kombucha led to a different rate of substrate consumption.

#### 3.3.3. Variations in Ethanol Content

**Figure 5:**
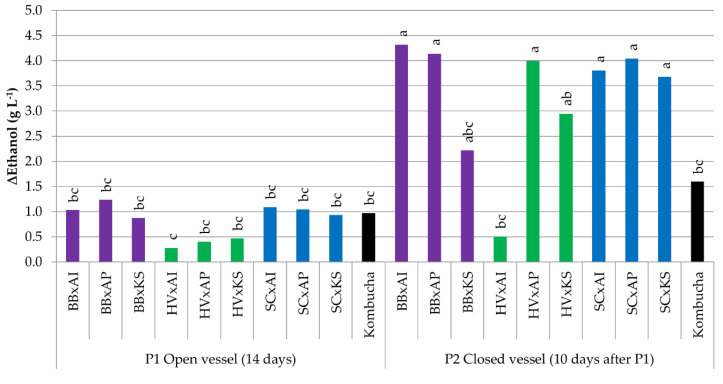
Difference in ethanol (g L^−^^1^) of samples between day 0 (after inoculation) and the end point (14 days for pure cultures, 14 days in an open vessel for P1, and 10 days in a closed vessel for P2 following P1). ANOVA was performed with α = 0.05 and *n* = 3. Common letters imply non-significant differences between means. Colors reflect the yeast species in the coculture. BB = *Brettanomyces bruxellensis* (purple), HV = *Hanseniaspora valbyensis* (green), SC = *Saccharomyces cerevisiae* (blue), AI = *Acetobacter indonesiensis*, AP = *Acetobacter papayae*, KS = *Komagataeibacter saccharivorans*, and “x” = a coculture.

The ethanol increase was lower at P1 (from +0.3 to +1.2 g L^−^^1^) than P2 because of the inhibition of oxidative metabolism with oxygen deprivation (from +0.5 to +4.3 g L^−^^1^) (Figure 5). The production of ethanol by cocultures involving *S. cerevisiae* at P1 was higher than those occurring in monocultures in open IC (+0.1 g L^−^^1^) (Table 3). This shows that this species in cocultures switched to fermentative metabolism, even in open IC. This switch also involved an increase in invertase activity due to the lower energetic yield of glucose consumed through fermentation compared to respiration (Figure 3). Between P1 and P2, the ethanol content increased significantly for BB x AI, BB x AP, HV x AP, and all cocultures involving *S. cerevisiae* (*p* < 0.05). The ethanol production of original kombucha was +1.0 g L^−^^1^ at P1, with no significant variation at P2. Most ethanol production was significantly higher in cocultures, except BB x KS, HV x AI, and HV x KS. This could suggest that biological ethanol oxidation is influenced by microbial dynamics. The presence of the pellicle in original kombucha could play a specific role in the enhancement of ethanol oxidation in particular.

#### 3.3.4. Variations in the Free Amino Nitrogen Content

**Figure 6:**
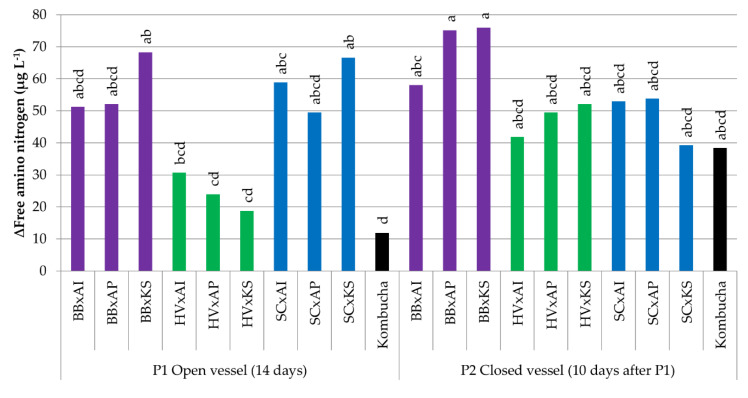
Difference in free amino nitrogen (µg L^−^^1^) of samples between day 0 (after inoculation) and the end point (14 days for pure cultures, 14 days in an open vessel for P1, and 10 days in a closed vessel for P2 following P1). ANOVA was performed with α = 0.05 and *n* = 3. Common letters imply non-significant differences between means. Colors reflect the yeast species in the coculture. BB = *Brettanomyces bruxellensis* (purple), HV = *Hanseniaspora valbyensis* (green), SC = *Saccharomyces cerevisiae* (blue), AI = *Acetobacter indonesiensis*, AP = *Acetobacter papayae*, KS = *Komagataeibacter saccharivorans*, and “x” = a coculture.

An increase of the FAN concentration between +19 and +80 µg L^−^^1^ was also observed in cocultures compared to sugared tea (initial average concentration of 63 µg L^−1^) (Figure 6). The FAN increase was significantly lower for HV x KS compared to BB x KS and SC x KS at P1 (*p* < 0.05). The FAN increase in original kombucha was 12 µg L^−^^1^ at P1 and was not significantly different compared to P2. The fact that FAN increases were similar between P1 and P2 suggests that oxygen deprivation could occur at P1 in cocultures because of the oxidative metabolism of AAB, as this behavior contrasts with that of yeast monocultures (Table 1).

#### 3.3.5. Acidification by the Production of Organic Acids

**Figure 7:**
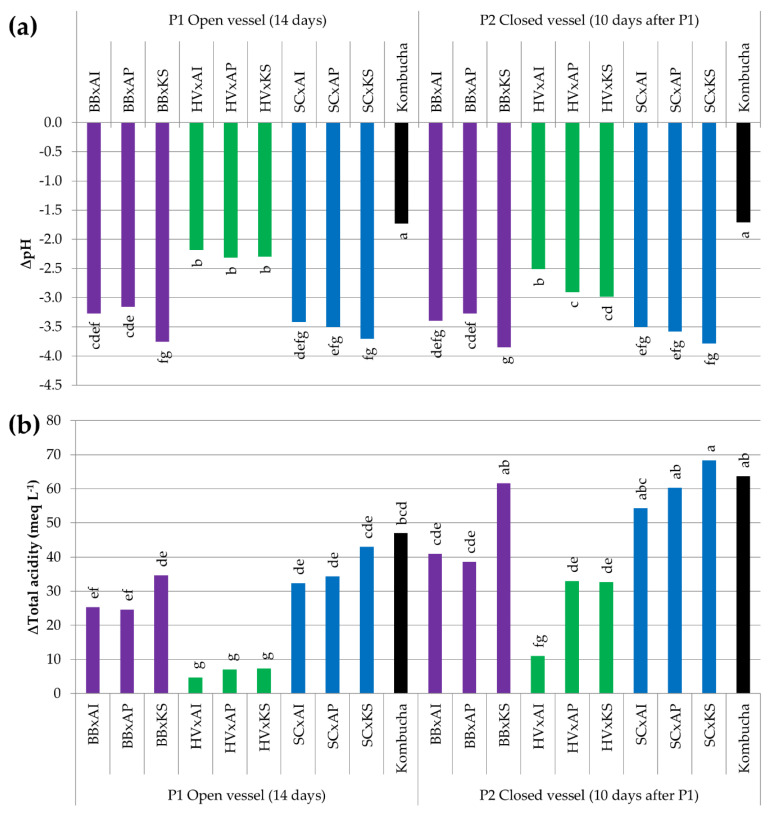
Difference in the (**a**) pH value (arbitrary unit) and (**b**) total acidity (meq L^−^^1^) of samples between day 0 (after inoculation) and the end point (14 days for pure cultures, 14 days in an open vessel for P1, and 10 days in a closed vessel following P1 for P2). ANOVA was performed with α = 0.05 and *n* = 3. Common letters imply non-significant differences between means. Colors reflect the yeast species in the coculture. BB = *Brettanomyces bruxellensis* (purple), HV = *Hanseniaspora valbyensis* (green), SC = *Saccharomyces cerevisiae* (blue), AI = *Acetobacter indonesiensis*, AP = *Acetobacter papayae*, KS = *Komagataeibacter saccharivorans*, and “x” = a coculture.

The pH of all cocultures dropped between −2 and −4 units (initial average of 6.64) and the total acidity increased from +4 to +70 meq L^−^^1^ (initial value < 1 meq L^−^^1^) (Figure 7). After 14 days, cocultures involving *H. valbyensis* displayed a drop of pH that was significantly weaker than the others (*p* < 0.05) and their average total acidity values were the lowest (*p* < 0.05). Between P1 and P2, a significative drop of pH could be observed for HV x AP and HV x KS only (*p* < 0.05). Moreover, the total acidity increased significantly for BB x KS, HV x AP, HV x KS, and all cocultures involving *S. cerevisiae*, which suggests that the oxidative metabolism of AAB was maintained after the closing of the vessel. This could have been allowed by the presence of residual oxygen after the closing of bottles.

The drop of pH of original kombucha remained the same at P1 and P2, with −1.7 units (average initial pH of 6.04). The increase of total acidity was +47 meq L^−^^1^ at P1 and +63.7 meq L^−^^1^ at P2, but these average values were not significantly different (average initial total acidity of 3 ± 1 meq L^−^^1^). It is noticeable that despite having one of the highest values for the total acidity, the pH decrease of original kombucha was less intense than all cocultures and supposes a stronger buffering capacity. While a consistent cellulosic biofilm was present in original kombucha, no consistent pellicle was visible for cocultures. Instead, cellulosic fragments were floating at the surface. Therefore, in the absence of a consistent pellicle, cocultures were able to produce organic acids in similar quantities to original kombucha, which implies that the biofilm is not necessary to complete the P1 phase.

The production of organic acids in cocultures and original kombucha is detailed in Table 3. No target organic acid could be detected in sugared black tea and among them, no citric acid was detected in any sample.

The main organic acids produced during P1 were acetic and gluconic acids, with the concentration increase ranging below +2.7 g L^−^^1^ and below +2.2 g L^−^^1^, respectively. The lowest increases were associated with cocultures involving *H. valbyensis*. These organic acids are mainly produced by AAB as a result of oxidative metabolism from ethanol and glucose. At P1, the gluconic acid concentrations of cocultures involving *H. valbyensis* were significantly lower than those of cocultures involving *B. bruxellensis* (*p* < 0.05). The results suggest that the production of organic acids reflects the capacity of the paired yeast to make these substrates available, as it has been established that *H. valbyensis* possesses poor fermentative and invertase activities. Between P1 and P2, no significant change in the concentration of acetic and gluconic acids occurred for most cocultures, except BB x KS and SC x KS, whose acetic acid concentrations increased from +7.3 to +12.7 g L^−^^1^ (*p* < 0.05). This indicates a capacity of *K. saccharivorans* to maintain the production of acetic acid in conditions of oxygen limitations compared to *Acetobacter* sp., possibly through a pathway other than oxidative metabolism [44,45]. Other organic acids, such as succinic acid, were detected in all cocultures except for BB x KS at P1 and BB x KS and SC x KS at P2, which suggests a link with the presence of *K. saccharivorans,* especially in oxygen limitation conditions. Malic acid was only detected in HV x KS at P1 and HV x AI and HV x KS at P2. Lactic acid was only detected in all cocultures involving *H. valbyensis*, which seems to characterize this species (Table 1). The links established between the production of malic, succinic, and lactic acids and the type of metabolism do not appear clearly for cocultures, probably due to the complexification of metabolic pathways in cocultures.

In original kombucha, an increase of acetic acid and gluconic acid production of +1.6 and +1.7 g L^−1^, respectively (initial average concentrations of acetic acid of 0.4 g L^−1^ and gluconic acid of 0.3 g L^−1^) and low production of lactic and succinic acids inferior to +0.2 g L^−1^ at P1 (lactic and succinic acids not detected at day 0) (Table 3) were observed. Qualitatively, the organic acid composition of kombucha gathers all organic acids detected in cocultures, with the exception of malic acid. On the contrary, lactic acid was detected and was characteristic of cocultures with *H. valbyensis*. Quantitatively, the original kombucha composition in acetic and gluconic matched those of all cocultures involving fermentative yeasts (excluding *H. valbyensis*) at P1, but at P2, this only applied to cocultures involving *Acetobacter* sp., since the presence of *K. saccharivorans* induced an intense production of acetic acid that was probably due to the intensification of ethanol through alcoholic fermentation induced by oxygen deprivation. Indeed, this phenomenon did not occur in cocultures including *H. valbyensis*.

The coculture results underline the impact of yeasts and AAB metabolism on the matrix and variation occurring between yeast and AAB species. The use of statistical treatment can help in visualizing the phenomenon occurring in such complex systems and determine the similarities with original kombucha.

#### 3.3.6. Principle Component Analysis as a Visual Tool for Understanding Complex Microbial Interactions

Principle Component Analysis (PCA) was performed on cocultures and original kombucha for P1 and P2 separately, in order to characterize the two different phases in terms of the impact on the chemical composition of the sugared tea matrix (microbial population data were excluded). Both PCA possessed a satisfactory sum of proper values with Dim1 and Dim2 axes (77.40% for P1 and 67.91% for P2).

**Figure 8:**
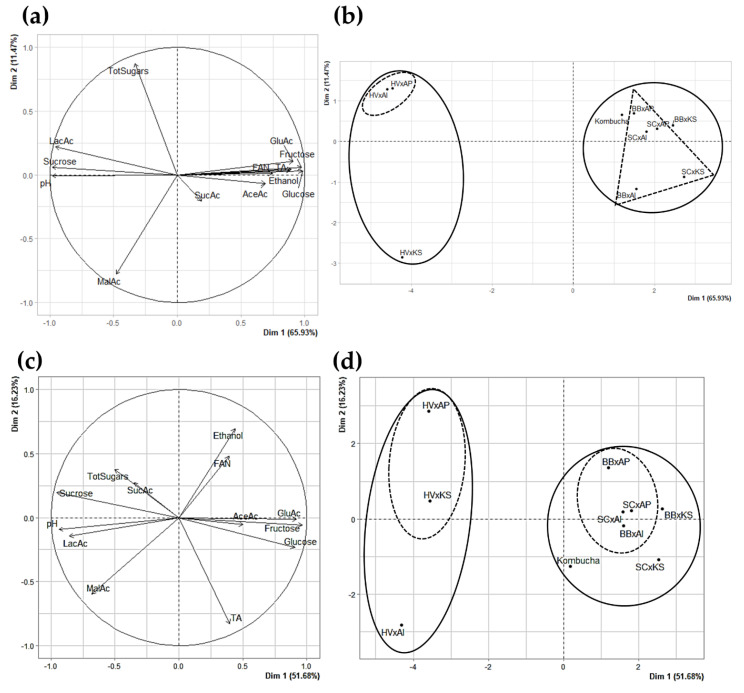
Principle Component Analysis of coculture and original kombucha samples using parameters of the chemical composition. (**a**) Parameter or vector plot for P1, (**b**) sample plot for P1, (**c**) parameter or vector plot for P2, and (**d**) sample plot for P2. Continuous circles gather samples of the same primary clusters and dashed line circles gather samples of the same sub-clusters according to hierarchical ascendant clustering analysis.

The parameter plot of P1 (Figure 8a) was primarily structured by the Dim1 axis positively characterized by the glucose, fructose, acetic acid, gluconic acid, ethanol, FAN concentrations, and total acidity (cos^2^ ≥ 0.7), and negatively by the pH, and sucrose and lactic acid concentrations (cos^2^ ≤ −0.7). Dim1 translated the biological acidification process targeted during P1 in an open vessel. This acidification (increase of total acidity and decrease of pH) was mainly caused by the production of acetic and gluconic acids by AAB. Their production was dependent on the release of monosaccharides (glucose and fructose) from sucrose that can efficiently be hydrolyzed by yeast invertase activity. Lactic acid production was associated with low invertase and fermentative activities, as observed in *H. valbyensis* or *S. cerevisiae* monocultures in open IC and in cocultures with *H. valbyensis* (Table 1 and Table 3). Dim 2 was positively characterized by total sugars concentrations and negatively characterized by malic acid, and translated differences in the profiles of organic acids from yeasts and the sugar consumption of cocultures. As a result, the sample plot (Figure 8b) coupled with hierarchical clustering discriminated cocultures with different levels. The predominant difference separated cocultures including *H. valbyensis* from other yeasts. This was caused by the low sucrose hydrolysis and fermentative capacity of *H. valbyensis* that prevented the access of AAB to monosaccharides and ethanol and thus efficient acidification of the medium. Secondarily, the clustering discriminated the cocultures, including *K. saccharivorans* and *Acetobacter* sp., for all coupled yeasts except *S. cerevisiae*. Original kombucha belonged to the cluster including BB x KS, highlighting the closeness in chemical compositions.

The parameter plot of P2 (Figure 8c) was mainly structured by Dim1, which was positively characterized by glucose, fructose, and gluconic acid concentrations (cos^2^ ≥ 0.7), and negatively characterized by the pH, and lactic acid and sucrose concentrations. Dim2 was positively characterized by the ethanol concentration and negatively characterized by the total acidity (cos^2^ ≤ −0.7). Specifically, Dim1 could be representative of the invertase activity and Dim2 of AAB activity (oxidation of ethanol and increase of the total acidity). It can be noticed that total sugar and ethanol are not anticorrelated, since, during P1 and even P2, the balance between total sugars and ethanol is biased by its conversion in acetic acid by AAB. The clustering (Figure 8d) again discriminated cocultures including *H. valbyensis* from the others and cocultures including *K. saccharivorans* from those with *Acetobacter* sp. (except for cocultures including *H. valbyensis*). Moreover, original kombucha again belonged to the cluster including BB x KS and SC x KS.

The comparison of parameter plots of P1 and P2 reflects the shift from the combined yeasts and AAB metabolisms, with a positive correlation of invertase activity, fermentation, and acidification during the acidification phase (all strongly contributing to Dim1) (Figure 8a), towards the prevalence of yeast metabolism (strong contribution to Dim1), with a lower correlation with AAB oxidative metabolism during the carbonation phase (ethanol and total acidity strongly contributing to Dim 2) (Figure 8c).

These results show that yeast metabolism was key throughout the process of fermentation: P1 required yeasts to hydrolyze sucrose and produce ethanol for their conversion into acetic and gluconic acids by AAB and P2 relied on fermentation to complete natural carbonation. Nevertheless, *K. saccharivorans* still strongly impacted the chemical composition during P2 because of its ability to maintain oxidative metabolism, especially when ethanol production is abundant, which was not as visible for *Acetobacter* sp. More generally, yeast metabolism is the main factor influencing the kombucha composition and the influence of AAB is secondary and mostly impacts the organic acid profile.

### 3.4. Sucrose Utilization Strategies as a Basis of Microbial Interactions in Kombucha

The experimental results obtained in this study confirm the metabolic interplay between yeasts and acetic acid bacteria that has been theorized for kombucha made from sugared black tea [17,49,51,52]. They highlight, for the first time, the key role of yeasts and their metabolisms in terms of invertase activity and the fermentation capacity, which can differ, depending on yeast strains (Table 1). Bibliographic resources and the NCBI database have reported the existence of genes encoding invertase in *S. cerevisiae* (internal and extracellular enzyme) [53,54,55,56] and *B. bruxellensis* (internal and extracellular enzyme) [57,58,59,60], but few data is available for *H. valbyensis*. However, genomic studies on the *Hanseniaspora* genus reported the loss of the *SUC* gene of the branch to which *H. valbyensis* belongs, thus supporting the low sucrose hydrolysis observed (Table 1) [61]. On top of glucose, fructose, and ethanol, yeasts may also release nitrogenous substrates through the conversion of bound amino-acids, such as proteins and peptides, into free amino acids, since the results suggest a correlation between FAN and fermentation (Figure 8a). Yeast can release free amino acid through extracellular proteolytic activity and/or autolysis [62,63,64,65], which could benefit AAB as well [66]. As a result, the metabolism of yeasts directly impacts the flux of available substrates for AAB. Nevertheless, AAB studied in this work were able to grow in sugared black tea without the presence of yeasts to hydrolyze sucrose, similar to the AAB strains used in Wang et al. (2020) [17]. From the perspective of the present results and the experimental conditions of the work cited before, the hypothesis of a snowball effect is supported: Initial spontaneous hydrolysis of sucrose in acidic conditions would be amplified by a pH drop resulting from organic acid production by AAB (Figure S3) [67]. It could even be hypothesized that such a phenomenon also applied for cultures involving *H. valbyensis* (Table 1, Figure 4 and Figure 7). However, the existence of enzymatic invertase activity in AAB is not to be excluded and more research needs to be carried out in terms of the metabolic pathways of AAB [68]. However, Balasubramaniam and Kannangara (1982) reported low invertase activity of *K. xylinus* and determined that sucrose consumption was achieved through the activity of sucrose phosphorylase [69]. In all cases, sucrose hydrolysis performed by yeasts except *H. valbyensis* was more efficient than that induced by AAB alone (Table 1). It should be underlined that besides oxidative metabolism, AAB can use monosaccharides through glycolysis, tricarboxylic acids (TCA), and pentose phosphate pathways [46]. The presence of acetic, succinic, and malic acids in AAB monocultures are markers of these metabolisms (Table 1), in agreement with coculture results. However, the metabolic activity of AAB mostly depends on the substrates released by yeast and the composition in AAB can impact the profile of produced organic acids (Table 3 and Figure 8).

The microbial dynamics of original kombucha after bottling (P2) were investigated for the first time and highlighted the microbial interaction resulting in the decrease in the population of *H. valbyensis* triggered by oxygen limitations (Figure 3). As *S. cerevisiae* exhibited more metabolic activity in monocultures and cocultures in oxygen-limited conditions, it can be hypothesized that a negative interaction with *H. valbyensis* could occur in original kombucha. *Saccharomyces*–non-*Saccharomyces* interactions have been studied and are not fully understood, but possible mechanisms include the production of toxic compounds [70,71,72,73], competition with other nutrients such as sugars or amino acids [74], cell–cell contact mechanisms [75], and flocculation [76]. It was observed that *S. cerevisiae*’s metabolism in cocultures switched to high invertase activity and fermentation during P1 (Figure 4 and Figure 5). This difference of behavior with the monocultures (Table 1) could be explained by an evolutionary mechanism in *S. cerevisiae*, often compared to “The Prisoner’s Dilemma”, which modulates its invertase activity according to the presence of “cheaters” without such enzymatic activity [13,77,78]. Therefore, the *SUC* gene coding for the invertase is kept at a minimum expression in monocultures, so that *S. cerevisiae* can hydrolyze sucrose progressively. A shift to a higher expression level can occur in the presence of other “cheater” species, leading *S. cerevisiae* to rapidly consume sugars to outgrow other populations and inhibit them through the release of toxic metabolites, such as ethanol [39]. However, in the case of kombucha, this benefits AAB because of the increase of oxidizable substrates and possibly through the increased availability of amino acids. Analytical data regarding oxygen consumption during kombucha fermentation is needed to assess its role in microbial interactions, namely taking into account the presence of the pellicle, which is thought to reduce the access to oxygen in the liquid phase [50,79]. It is worth noting that the pellicle was not necessary for AAB in cocultures to perform efficient organic acid production, thus raising questions about additional functions other than facilitating oxygen access to AAB during kombucha fermentation [50].

Differences between cocultures and kombucha also appeared in the chemical composition of the liquid phase. Some kinetics in original kombucha seemed to be intermediate compared to cocultures, especially with sucrose hydrolysis at P1 (Figure 3), the ethanol concentration at P2 (Figure 5), and the FAN concentration (Figure 6). This could be explained by the balance in the yeast population, namely between *B. bruxellensis* and *H. valbyensis*, which could lead to a leveling of global yeast metabolism during original kombucha fermentation (Figure 3). The role of *H. valbyensis* remains enigmatic regarding the technological aspect of kombucha fermentation. As the present study did not explore volatile compounds, it cannot be excluded that this yeast strain could contribute to the olfactive profile of kombucha beverages. More subtle microbial interactions could also take place in terms of amino acid consumption or chemical signaling between *H. valbyensis* and other yeasts and bacteria [74]. Overall, the results point to an eco-evolution in kombucha between species that possess and do not possess *SUC* genes necessary for the use of sucrose as a substrate. *H. valbyensis* and AAB would then be labeled as “cheaters” by taking benefits from the invertase activity of other yeasts, such as *B. bruxellensis* and *S. cerevisiae*, without contributing themselves to the production of public goods (here, monosaccharides) [80,81]. However, it has been reported in *S. cerevisiae* x *Escherichia coli* cocultures that, by lowering the population of public good producers (*S. cerevisiae*) at high population levels, cheaters (*E. coli*) could consequently stabilize the sucrose hydrolysis rate, thus maintaining both population levels [78]. A similar phenomenon could also have taken place in the present study, since lower yeast populations in cocultures with AAB compared to monocultures were also observed (Figure 1 and Figure 2). *H. valbyensis* and AAB could therefore contribute to the stability of the community, as it was reported that *Hanseniaspora* sp. could achieve fast growth due to the evolutionary loss of cell-cycle components, thus limiting nutrient access [82], and acidification of the medium by AAB induced the selection of acidophilic species [83].

## 4. Conclusions

Microbial interactions occurring in kombucha have often been described as “symbiotic”, which does not imply mutual benefits, as described by the word “mutualistic”, but rather that all species manage a stable coexistence, possibly through interactions that are yet to be defined [1,13]. Although the present study lacks an exhaustive insight into the volatile and non-volatile metabolites, it was possible to characterize nutritional interactions. As AAB are not dependent on yeasts to access substrates, it appears that yeast–AAB interactions display a non-strict parasitism relationship (as sucrose hydrolysis occurred in AAB monocultures and was sufficient to increase their population at the same level as in original kombucha). No evidence of a beneficial nutritional interaction for yeast could be determined. There is also evidence that AAB induce the production of substrates by *S. cerevisiae* by indirectly rerouting its metabolism, thus enhancing the benefits of this parasitic relationship. Moreover, sucrose appears to play a central role in kombucha symbiosis not only in yeast–bacteria interactions, but also potentially between yeast species. While the AAB composition secondarily impacts the profile in organic acids, the yeast composition in kombucha consortia is crucial for the global microbial dynamics, the chemical composition of the beverage, and the sensory characteristics of the product [9].

This study only includes one kombucha consortium, but many different microbial compositions exist. Nevertheless, many research works have focused on the identification of yeast and bacteria genera and it could be interesting to determine if the yeast profiles include genera or species with variable invertase activities and fermentative capacities. In silico studies relying on genome and protein databases combined with the results of past identification works of kombucha consortia could highlight the existence of patterns in yeast compositions in terms of links between species and activities. Yeast–yeast interactions in the context of kombucha fermentation should also be addressed in future research works, now that the importance of their role has been uncovered.

## Figures and Tables

**Table 1:** Change in the chemical composition of sugared black tea by pure cultures of yeast and bacterial strains isolated from black tea kombucha after 14 days in open and closed conditions of incubation.

Microorganism	*B. bruxellensis*		*H. valbyensis*		*S. cerevisiae*		*A. indonesiensis*		*A. papayae*		*K. saccharivorans*	
Incubation Condition	Open Vessel	Closed Vessel	Open Vessel	Closed Vessel	Open Vessel	Closed Vessel	Open Vessel	Closed Vessel	Open Vessel	Closed Vessel	Open Vessel	Closed Vessel
∆Total sugars (g L^−^^1^) *	−10.7 ^a^	−10.6 ^a^	−2.2 ^a^	−9.4 ^a^	0.0 ^a^	−7.9 ^a^	−3.6 ^a^	−0.9 ^a^	−0.7 ^a^	0.0 ^a^	−6.2 ^a^	−10.0 ^a^
∆Sucrose (g L^−^^1^)	−68.3 ^d^	−68.3 ^d^	−2.2 ^a^	−15.4 ^ab^	−36.1 ^c^	−68.3 ^d^	−6.9 ^ab^	−0.9 ^a^	−1.0 ^a^	0.0 ^a^	−12.3 ^ab^	−19.6 ^b^
∆Glucose (g L^−^^1^)	27.0 ^a^	26.9 ^a^	0.0 ^d^	0.9 ^d^	18.9 ^b^	28.5 ^a^	2.1 ^d^	nd	nd	nd	4.2 ^d^	9.0 ^c^
∆Fructose (g L^−^^1^)	29.8 ^a^	30.8 ^a^	0.0 ^d^	5.0 ^c^	17.2 ^b^	31.8 ^a^	1.1 ^cd^	nd	0.2 ^d^	0.4 ^d^	1.9 ^cd^	0.6 ^d^
∆Ethanol (g L^−^^1^)	3.2 ^a^	2.5 ^b^	0.1 ^d^	1.5 ^c^	0.4 ^d^	2.4 ^b^	nd	<0.1	nd	nd	nd	<0.1
∆Free amino nitrogen (µg L^−^^1^)	37 ^a^	52 ^a^	7 ^b^	31 ^ab^	3 ^b^	54 ^a^	3 ^b^	30 ^ab^	8 ^b^	29 ^ab^	8 ^b^	28 ^ab^
∆pH	−2.13 ^cd^	−1.86 ^cd^	−1.07 ^b^	−2.26 ^cd^	−0.38 ^a^	−1.67 ^c^	−2.64 ^d^	−2.25 ^cd^	−2.05 ^cd^	−2.04 ^cd^	−2.61 ^d^	−2.50 ^d^
∆Total acidity (meq L^−^^1^)	8.0 ^abc^	6.3 ^abc^	2.0 ^c^	6.7 ^abc^	1.0 ^c^	4.7 ^bc^	11.0 ^abc^	19.3 ^a^	7.0 ^abc^	5.7 ^bc^	19.3 ^a^	16.7 ^ab^
∆Acetic acid (g L^−^^1^)	0.38 ^a^	0.25 ^a^	nd	<0.1	<0.1	nd	0.25 ^a^	0.49 ^a^	0.28 ^a^	0.38 ^a^	0.40 ^a^	0.56 ^a^
∆Gluconic acid (g L^−^^1^)	<0.05	<0.05	<0.05	<0.05	<0.05	<0.05	0.91 ^a^	0.11 ^b^	0.56 ^ab^	0.18 ^b^	1.94 ^a^	0.12 ^b^
∆Lactic acid (g L^−^^1^)	nd	nd	0.46 ^a^	0.20 ^abc^	0.32 ^ab^	nd	nd	nd	nd	nd	<0.1	nd
∆Succinic acid (g L^−^^1^)	1.27 ^a^	1.19 ^a^	0.11 ^c^	0.76 ^b^	0.34 ^c^	1.27 ^a^	<0.1	<0.1	nd	nd	0.14 ^c^	0.16 ^c^
∆Malic acid (g L^−^^1^)	0.92 ^a^	1.4 ^a^	nd	1.00 ^a^	nd	1.24 ^a^	nd	0.22 ^b^	<0.1	0.18 ^b^	nd	<0.1

*: ∆Concentration = ∆Endpoint concentration − ∆Initial concentration; nd = not detected; common letters imply non-significant differences between average values (ANOVA test with α = 0.05 and *n* = 3).

**Table 2:** “Yeast x Acetic acid bacteria” couples used for cocultures.

Cocultures	*Acetobacter indonesiensis*	*Acetobacter papayae*	*Komagataeibacter saccharivorans*
*Brettanomyces bruxellensis*	BB x AI	BB x AP	BB x KS
*Hanseniaspora valbyensis*	HV x AI	HV x AP	HV x KS
*Saccharomyces cerevisiae*	SC x AI	SC x AP	SC x KS

**Table 3:** Difference in the organic acid content of samples between day 0 (after inoculation) and the end point (14 days for pure cultures, 14 days in an open vessel for P1, and 10 days in a closed vessel following P1 for P2).

Fermentation Phase	Coculture	∆Acetic Acid (g L^−1^)	∆Gluconic Acid (g L^−1^)	∆Lactic Acid (g L^−1^)	∆Succinic Acid (g L^−1^)	∆Malic Acid (g L^−1^)
P1 Open vessel (14 days)	BB x AI	0.7 ^bcd^	1.6 ^abc^	nd	0.3 ^abc^	nd
	BB x AP	0.6 ^bcd^	1.4 ^abcd^	nd	0.3 ^abc^	nd
	BB x KS	2.7 ^bc^	1.7 ^ab^	nd	nd	nd
	HV x AI	nd	0.1 ^ef^	0.3 ^a^	0.2 ^bc^	nd
	HV x AP	0.2 ^cd^	0.1 ^f^	0.4 ^a^	0.2 ^abc^	nd
	HV x KS	0.4 ^c^	<0.05	0.2 ^bc^	0.2 ^abc^	0.3 ^a^
	SC x AI	1.0 ^bcd^	1.2 ^abcde^	nd	0.3 ^abc^	nd
	SC x AP	0.8 ^c^	2.2 ^a^	nd	0.3 ^abc^	nd
	SC x KS	2.7 ^bcd^	1.3 ^abcde^	nd	0.4 ^abc^	nd
	Kombucha	1.6 ^bcd^	1.7 ^ab^	0.1 ^c^	0.2 ^abc^	nd
P2 Closed vessel (10 days after P1)	BB x AI	2.0 ^bcd^	1.6 ^abc^	nd	0.6 ^abc^	nd
	BB x AP	1.6 ^bcd^	1.2 ^abcde^	nd	0.7 ^ab^	nd
	BB x KS	12.7 ^a^	2.0 ^a^	nd	nd	nd
	HV x AI	0.7 ^cd^	0.3 ^def^	0.2 ^bc^	0.7 ^ab^	1.0 ^a^
	HV x AP	2.4 ^bcd^	0.5 ^cdef^	0.1 ^cd^	0.7 ^ab^	nd
	HV x KS	2.9 ^bcd^	0.4 ^def^	0.3 ^ab^	0.5 ^abc^	0.2 ^a^
	SC x AI	2.2 ^bcd^	2.3 ^a^	nd	0.8 ^a^	nd
	SC x AP	2.2 ^bcd^	2.4 ^a^	nd	0.8 ^a^	nd
	SC x KS	11.3 ^a^	2.2 ^a^	nd	nd	nd
	Kombucha	2.1 ^bcd^	0.8 ^bcdef^	0.1 ^cd^	0.3 ^abc^	nd

ANOVA was performed with α = 0.05 and *n* = 3, and common letters imply non-significant differences between means. BB = *Brettanomyces bruxellensis*, HV = *Hanseniaspora valbyensis*, HO = *Hanseniaspora opuntiae*, SC = *Saccharomyces cerevisiae*, AI = *Acetobacter indonesiensis*, AP = *Acetobacter papayae*, KS = *Komagataeibacter saccharivorans*, and “x” = a coculture.

## Supplementary Materials

The following are available online at https://www.mdpi.com/2304-8158/9/7/963/s1, Figure S1: Sum-up diagram of the experiment, Table S2: Description of research samples with abbreviations, Figure S3: Hypothetical “snowball effect” explaining sucrose hydrolysis by acetic acid bacteria.
